# Sandplay Therapy and Active Imagination: What Are the Similarities and Differences? Reflections about Jung’s Writings on Active Imagination

**DOI:** 10.3390/bs14070553

**Published:** 2024-06-29

**Authors:** Yura Loscalzo

**Affiliations:** Department of Health Sciences, School of Psychology, University of Florence, 50135 Florence, Italy; yura.loscalzo@gmail.com

**Keywords:** active imagination, Jung, Kalff, psychoanalysis, sandplay therapy, sandtray

## Abstract

Jung stated that active imagination is a fundamental component of the second phase of an analysis that can continue even outside the analytic setting. Since it can be conveyed through various expressive techniques, such as writing, drawing, and painting, it is possible to argue that all forms of psychotherapy based on art (e.g., poetry, dance, and theater) originate from Jung’s contribution about active imagination. This paper focuses on Sandplay Therapy as one of the forms of expression rooted in active imagination. Apart from some critical differences between the two analytic processes (e.g., active imagination is usually prompted in the last phase of the analysis, while Sandplay Therapy might be used since the first sessions), there are several convergences. Among the principal analogies, consciousness lends its expressive means to the unconscious, which decides what to depict. Also, the resulting image is determined from both the consciousness and the unconscious and is related to the person’s conscious situation. Finally, I suggest that Sandplay Therapy—aside from contributing to the subsequent development of active imagination itself (as suggested by Dr. Carducci)—might also be used to practice active imagination in a “facilitated” and protected setting. It would help let the unconscious come up while creating the image in the sandtray, and it fosters the confrontation between the unconscious and the consciousness through the contemplation of the image in the sandtray.

## 1. Introduction

Active imagination is an introspective method created by Jung to favor the observation of inner image flow, and it is based on suspending the critical judgment of consciousness [1]. De Luca Comandini [2] specified that active imagination constitutes—at an imaginal level—a close encounter between the Ego and the unconscious. Unlike a dream, the consciousness and the unconscious are involved simultaneously and in the same imaginal scene during active imagination. Consequently, it can trigger a fluid interchange between the consciousness and the unconscious [2]. Furthermore, the products that emerge during active imagination are more defined than dreams since they arise in a conscious mental state [3]; the consciousness observes the unconscious content in a waking state rather than asleep [1]. It is essential to emphasize that active imagination is also different from fantasy. Fantasy is a product of consciousness, a person’s invention. In active imagination (if consciousness does not interfere), images have their own life, and symbolic events develop according to their logic, i.e., by focusing on the mental image, there is a transformation, and it comes to life [3].

In brief, the method of active imagination—as described by Jung to Mr. O—requires selecting an image and contemplating it, observing how it begins to transform without trying to do something to prompt the change in the image. Then, once the image comes to life, the person must enter the scene and talk (at an imaginal level) with the figures that might have arisen in the image [4]. Therefore, there are two critical phases, as recently summarized by Loscalzo [5] in her thorough presentation of active imagination as a technique employable across different psychodynamic approaches. First, the patient identifies a starting stimulus (e.g., an image of a dream), and he/she then focuses on it—in a void of consciousness—until it comes to life. Then, the consciousness actively participates in understanding the image (both intellectually and emotionally) to implement the gained insight in life. Given that active imagination requires a dialogue between the Ego and the unconscious, Jung [6] warns that when the patient’s Ego is not strong enough to face the unconscious, it is advisable to consolidate the Ego instead of fostering the understanding and assimilation of the products of the unconscious. Moreover, active imagination is generally conducted alone, without the analyst’s presence [7]. Jung, in fact, considered the practice of active imagination as proof of the patient’s psychological maturity and willingness to become independent from the analyst [2,7]. Finally, the analyst usually does not provide interpretations of the material shown by the patients. Jung, even if he acted as a mentor in some cases (e.g., suggesting using bright colors), avoided interpretations as much as possible, and (when interpreting) he tried to be as hypothetical as possible, never going beyond the image itself [7].

Active imagination can express itself in various ways; Jung himself used different expressive techniques, such as writing, drawing, and painting, to give symbolic form to his experiences. Thus, Chodorow [7] suggested that it is possible to argue that all forms of psychotherapy based on art in its different expressions (for example, poetry, dance, and theater)—and also Sandplay Therapy—originate from Jung’s contribution to active imagination.

The present paper focuses on (Kalff) Sandplay Therapy and its connections with active imagination, in line with Jungian analysts who suggested that Sandplay Therapy might be conceived as one of the expressive forms through which active imagination might be conveyed [7,8,9]. More specifically, besides illustrating the 1999 congressional contribution by Paola Carducci (who was a registered Jungian analyst and founding member of the Italian Association for Sandplay Therapy), I will discuss a personal reading of Jung’s writings on active imagination—as collected by Johan Chodorow [7]—by adopting the perspective of Sandplay Therapy and thus underlining the points sustaining the resemblances (and the differences) between the two analytic processes.

It is crucial to highlight that this paper pursues the objectivity required by the internationally recognized standards for scientific (theoretical) papers. Therefore, even if the differences and similarities between Sandplay Therapy and active imagination are presented as personal thoughts, they are grounded in a thorough reading of Jung’s writings on active imagination and publications by Jungian/Kalff Sandplay Therapy analysts.

## 2. Sandplay Therapy: Similarities and Differences with Active Imagination

### 2.1. Paola Carducci’s Congressional Contribution

Paola Carducci was a recognized Jungian analyst and founding member of the Italian Association for Sandplay Therapy. In 1999, during a congress (whose contributions were published in 2002 as a book) held in Rome and devoted to active imagination and its different forms of expression, she presented Sandplay Therapy as one of the forms of expression rooted in active imagination [8]. It is important to underline that the three recognized roots of Sandplay Therapy are the World Technique by Margaret Lowenfeld [10], Jungian analytical psychology—generally, without a specific reference to active imagination—and Eastern contemplative traditions [11].

First, Carducci [8] pointed out that those who practice Sandplay Therapy in line with Dora Kalff conceive it as *a therapeutic modality connected to active imagination but at the same time distinct from it*. Hence, she presented both the divergences and convergences between Sandplay Therapy and active imagination.

The fundamental difference between the two analytical processes is that active imagination is used in the last, self-analytical phase of analysis [8]. Since active imagination fosters the confrontation with the unconscious (in the absence of the analyst), it requires that good analytical work has previously taken place so that the dialogue between the consciousness and the unconscious is denoted by equal dignity (i.e., the unconscious does not overwhelm the consciousness and the consciousness does not devalue the unconscious) [2]. Sandplay Therapy might be used from the beginning of the analysis since it foresees the physical presence of the analyst, who has a fundamental role in protecting the patient [8]. In line with the different stages in which Sandplay Therapy and active imagination are implemented, a further aspect of divergence is related to the transferential dynamic. In Sandplay Therapy, the analyst is physically present—albeit placed at a slight distance from the patient and he/she does not interpret—since the analyst plays the (fundamental) role of containing and protecting the patients and their productions. Instead, as active imagination is generally reserved for the final part of the analysis (when the analysand has greater self-awareness and has developed greater independence from the relationship with the analyst), the role of the analyst is no longer as relevant as in Sandplay Therapy and can be absent [8]. The role of the analyst in Sandplay Therapy is critical, also considering the importance recognized by Dora Kalff to the relationship between the therapist and the client, that is, to transfer and counter-transfer, or to co-transfer, as suggested by the Jungian analyst Kay Bradway [12]. As Martin Kalff [12] pointed out, some figures in the sandtray might even represent the qualities the analysand attributes to the analyst.

Regarding the aspects that Sandplay Therapy and active imagination have in common, Carducci [8] underlined that the analyst does not impose nor suggest the patient’s imagination, that is, *the analysands are entirely free in their expression*. The analyst selects the miniatures and the objects displayed on the shelves to represent the reality of the psyche and its symbols. However, they only constitute an evocative stimulus (and an aid) for the representation of the analysand. In line with this freedom left to the patient, Carducci [8] underlined the importance of including plastic materials (e.g., clay) among the shelves, in addition to the various miniatures, to allow people to build objects suited to their fantasies if they feel it is necessary. In summary, the analyst (as in active imagination) is not directive or intrusive and does not choose images with healing power for the patient. The analyst chooses images with evocative power and displays them on the shelves, leaving the patient free to express his/her imagination.

Another similarity relates to *interpretation* since the analyst is present and observes the inner images represented by the person in the sandtray but does not interpret them. The analyst takes a photo at the end of the session to document the analytic process; however, it is only at the end of the analytic process that the therapist and the patient look together at the images made in the sandtray during the therapy (although this is not a practice shared by all analysts using Sandplay Therapy). Therefore, during the session with sandplay, the analyst does not verbalize his/her (possible) interpretations; the interventions consist instead of comments or amplifications of the symbols. The analytic process activated through Sandplay Therapy takes place at a level much closer to the unconscious than verbal analysis; consequently, it is essential to avoid disturbing the analytic process through early interpretations and conceptualizations since they could lead to the awareness of contents that must instead still be kept inside by the analysand, to be elaborated at a later time when he/she is ready [8]. Furthermore, Dora Kalff urged the analyst *to put aside his/her theoretical system* to allow the patient to express himself/herself freely. She therefore recommended (in addition to not intervening and making judgments) participating in the analysand’s experience by creating a void within oneself (typical of a Buddhist practice) and maintaining a spiritually active attitude (or, using Jungian terminology, maintaining active the analysist’s transcendent function), thus fostering the possibility of psychic transformation in the patient. Therefore, the attitude suggested by Dora Kalff corresponds to Jung’s attitude towards the images his patients brought during their analyses (not only the images that arose from active imagination) [8].

A further point that Sandplay Therapy has in connection with active imagination relates to *the images that emerge* from the unconscious. Carducci [8] underlined that the images in the sandtray are not always aesthetically beautiful—as often happens with active imagination’s images—given that the unconscious can also express itself using archaic language.

Finally, it is interesting to report Carducci [8]’s suggestion about the interchangeable relationship between Sandplay Therapy and active imagination. Sandplay Therapy, although originating from active imagination, can, in turn, contribute to its development. According to Carducci [8], the analytic process carried out with Sandplay Therapy—favoring the acquisition of the ability to represent images in front of the analyst—*readies the person to the following imaginative activity independent of the analyst*, namely, to active imagination as described by Jung.

### 2.2. Jung’s Writings on Active Imagination from the Perspective of Sandplay Therapy

In Jung’s writings regarding active imagination (including his self-analysis experience reported in his autobiography) collected by Johan Chodorow [7], it is possible to highlight some assertions that show points of contact between active imagination and Sandplay Therapy.

First, I suggest that the statement “often the hands know how to solve a riddle with which the intellect wrestled in vain” (par. 180) [13] might be used as the “manifesto” of Sandplay Therapy. In line with this assertion, Jung [14] further pointed out that when there is a high blockage at the conscious level, *only the hands are capable of fantasy*, i.e., they draw or model images that are sometimes alien to the conscious mind. The resemblance with Sandplay Therapy is that it involves using hands: they shape the sand, choose the objects, and place them in the sandtray; moreover, they often give life to images beyond the consciousness’ control. Dora Kalff [11] writes that the figures created in the sandtray can be conceptualized as three-dimensional representations of a psychic situation that allow an internal conflict (which could be challenging to verbalize [15]) to be made visible.

Furthermore, in his presentation of the individuation process of Miss X, Jung wrote that (during the creation of the mandalas) the figure seemed to develop on its own, often in opposition to conscious intentions [6]. In another writing, Jung [16] underlined how guiding the pencil and the brush during the painting or the foot during the dance was a dark and internal impulse, namely, an unconscious element that emerged in a pliable form. Jung also specified that “consciousness puts its media of expression at the disposal of the unconscious content” (par. 178) [13]. In sum, the product of active imagination (i.e., the new/third element created from this experience, or the symbol) originates from the union of conscious and unconscious elements since the confrontation between them creates a tension of energy that leads to the formation of a new element, which allows one to reach a new level of existence [13]. Consequently, during active imagination, the person notices that emerging figures always have a relationship with the conscious situation [17]. Hence, Jung affirms that *it is not the consciousness determining what to depict but rather the unconscious*. Moreover, *the resulting product is determined by both the consciousness and the unconscious and is related to the conscious situation of the person*.

I suggest this is also a peculiar experience of Sandplay Therapy, where the consciousness (through the hands, the sandtray, and the objects) loans its expressive tools to the unconscious content; namely, the Ego qualifies as an emissary of the Self [18]. Hence, even in Sandplay Therapy (like in active imagination), the person might undergo an experience whereby the image in the sandtray seems to originate on its own, even in opposition to conscious intentionality, given that consciousness lends its expressive instruments (such as the hands and the use of the sandtray and objects) to allow for unconscious contents to be represented [18,19]. Moreover, the symbols used in Sandplay Therapy (miniatures and objects, but also figures created in the sand) might have a relationship with the conscious situation of the person and represent the third element originating from the confrontation between the conscious and the unconscious. Jung [14] gave great value to the symbol as the individuation process cannot occur in its absence and since the unconscious can only be reached and expressed through symbols. Sandplay Therapy using miniatures (but also figures created in the sand without objects), namely objects/signs that may become living symbols in the field created by the analysand and the therapist, therefore constitutes a valuable means for the unconscious expression and the individuation process.

Sandplay Therapy could represent an effective way of representing contents that are not expressible verbally, in line with Jung’s [20] statement that patients try with all their energies to *give shape to the unspeakable*. Also, it might facilitate the distinction between the consciousness and the contents of the unconscious. This differentiation can occur through the *isolation of the unconscious by its personification*, and it is necessary to allow the consciousness to establish a relationship with it during active imagination [21]. Regarding the differentiation between the Ego and the figures of the unconscious, Jung [22] also specified that it is fundamental that the person *lives the images* instead of understanding and interpreting them.

I suggest a matching between these elements and Sandplay Therapy since the latter—thanks to the sandtray and the objects arranged on the shelf—can help the patients shape their inexpressible interiority and live it as happens during active imagination. More specifically, through the creation of images in the sandtray, the person gives a tridimensional form to his/her unconscious contents (or personifies them); furthermore, thanks to the observation/contemplation of the image, dialogue between the consciousness and the unconscious might take place (that characterizes active imagination). Sandplay Therapy facilitates the representation and experience of unconscious images due to the three-dimensionality provided by miniatures, the manipulation of the sand, and the use of the tray’s blue space. In line with this suggestion, Marinucci [15] advocated that emotions, affections, and memories that might not be expressed verbally could emerge and be elaborated in the sandtray.

Jung [3] also underlined the *importance of archetypes’ objectification* (which occurs through active imagination) since non-contained archetypes could flood the consciousness and lose their positive value in fostering psychic development. The closeness with Sandplay Therapy lies in the fact that the sandtray, concurrently constituting a free and protected space [11], allows for limiting of the risk of inflation by the unconscious. Furthermore, the images produced in the sandtray make it possible to objectify the products of the unconscious (also thanks to the use of miniatures) thus further limiting the risk of non-containment of the unconscious.

Another aspect concerns Jung’s consideration [20] about the *importance of doing rather than just talking*, which is why he encouraged his patients to paint and draw. He also adds that *creating a form* is critical as it *prompts the person to continuously study all its parts*, favoring the development of the image’s potential to its maximum [20]. In this vein, Sandplay Therapy allows the person “to do”, as the analysands move with their whole body as they turn around the shelves and use their hands while placing, removing, or moving objects in the sandtray. Furthermore, thanks to the production of the images in the sandtray, the person is stimulated to observe the image he/she created from various angles, fueling the development of its individuation potential. About the unfolding of the image’s potential to its fullest—albeit not referring in this case to active imagination, but to dreams (which can be used as a starting point for the practice of active imagination)—Jung [20] underlined that *if the person meditates long enough* upon a dream, carrying it inside him/herself and looking at it several times, *something almost always emerges*. I suggest this also happens when the person, having finished his/her picture in the sandtray, observes it in a meditative/spiritual state. Furthermore, even once the session is over, the analysands carry the image of what was created in the sandtray within them and can continue to observe and contemplate it inwardly.

Concerning the products of active imagination, Jung [3] writes that when the person concentrates on an internal image and does not interrupt its natural flow, *the unconscious produces a series of images that make up a complete story*. It also happens in the analytic process with Sandplay Therapy, which narrates the psychic development that turns towards the individuation of the analysand; sometimes, this narrative is evident, such as when the person uses the same miniature in several significant sand pictures. For instance, a 12-year-old boy placed a downed aircraft in his first sandtray (according to Dora Kalff, as an expression of his desperate condition); then, in his last sandtray (following the awakening of his energies), he positioned a helicopter ready to take off in the same place [11].

A further element of convergence between active imagination and Sandplay Therapy concerns interpretation. Jung [16] argued that *interpretations should be kept to a minimum*. When verbalized, they should never go beyond the confines of the image produced by the analysand, and they should be interspersed with innumerable “maybes”, “ifs”, and “buts”. For Jung [16], the image does not need interpretation; as it takes shape, its meaning becomes apparent. Hence, for Jung, it is unnecessary to make interpretations as the image carries its meaning. Similarly, Dora Kalff [11] argued that the analyst must interpret for him/herself the symbols emerging in the sandtray, and that (generally) it is not necessary to communicate what has emerged from the analyst’s reflections. Moreover, her son, Martin Kalff [23], took up exactly Jung’s [16] above-mentioned quote to suggest the respectful approach that the analyst should take in interpreting the images in the sandtray.

Finally, Jung’s autobiography—specifically, the section about his confrontation with the unconscious—contains some elements allowing a parallel between active imagination and Sandplay Therapy to be drawn. First, Jung [21] recounted that during his village-building game (which could be compared to building a village on a sandtray), he hesitated to build the altar he felt was needed in the church he had built. However, during a walk, he later found a red stone in the shape of a pyramid and *understood that it must be his altar*. I suggest that Jung’s experience resembles the choice of the objects on the shelves in Sandplay Therapy since the person can feel that he/she is “chosen by the object” rather than “choosing the object”, like Adorisio [24] distinguished between the experience of “letting oneself move”—typical of active imagination—and “moving”, and as Jung [21] reported that he felt that it was the appropriate stone.

Jung [21] also reported that, once he placed the red stone on the altar, he remembered the underground erect penis he saw in his dream as a child (the first dream he remembers), that is, through this symbolic game, he *contacted one of his most profound complexes* [7] and felt relief. It might be compared to placing an object (or creating a shape) in the sandtray, as this might activate unconscious elements and complexes and therefore a sense of relief. Jung [21], continuing the narration of his self-analysis experiment, proclaimed that thanks to his confrontation with the unconscious he understood the *therapeutic importance of discovering the hidden image behind an emotion*. In this vein, Sandplay Therapy allows the person to observe (in an objectified form) the images emerging from the unconscious, with the consequent uncovering of the image hidden behind the emotion.

In conclusion, since active imagination and (Kalff) Sandplay Therapy are two connected but distinct therapeutic modalities [8], it is important to underline a significant difference that arose from Jung’s writings about active imagination. Jung [3] stated that if the patient brings the images produced during active imagination to the analyst, he/she should accept them in a copy and leave the original with the patient. According to Jung [3], the person needs to relate to the image produced since he/she feels that the unconscious has been expressed in the image. On the other hand, Sandplay Therapy (generally) foresees that the analyst photographs the image created in the sandtray only once the patient has left the room, not urging the person to take a photo and not giving him/her a copy of the photo taken. However, it is essential to underline that the analysands observe and contemplate their image during the session in which they created it and that they can continue to observe it internally after having left the room of the analyst.

## 3. Conclusions

This paper highlighted that Sandplay Therapy might be conceptualized as a form of expression of active imagination—with which it then shares many similarities—in line with previous analysts’ assertions [7,8,9]. Dora Kalff developed Sandplay Therapy thanks to Jung’s personal and theoretical support. Jung suggested she conduct her analysis with Emma, his wife. Moreover, given her predisposition to work with children and adolescents, Jung advised her to go to London to meet Margaret Lowenfeld. Thanks to this encounter, Dora Kalff learned the World Technique, which she later developed within the Jungian framework of the individuation process [25]. Consequently, although there are some differences between active imagination and Sandplay Therapy (as they are two different analytic processes), many points of convergence also arise.

Differences are mainly about the phase in which they are generally implemented. Active imagination is reserved for the last phase of the analysis (although it can sometimes be used in the initial phases of therapy to proportion the unconscious activity). In contrast, Sandplay Therapy might be used from the first sessions. Furthermore, the analyst is usually absent in active imagination while he/she is present, acting as a support and container, in Sandplay Therapy [8]. In addition, I proposed a difference regarding the product/image arising from the two therapeutic processes. Jung [3] recommended that the patient takes the original (for example, a painting). Sandplay Therapy, in contrast, foresees that the analyst photographs the patients’ images after they leave the room. Hence, the analysands do not carry a photo of what they created in the sandtray (although they may keep the image of it internally).

As for analogies, there are many. Carducci [8] reported, as primary similitudes, that the analysand is entirely free in his/her expression and that the analyst does not verbally interpret the images in both analytic processes. I also proposed other likenesses between the two methods by referring to Jung’s writings on active imagination and his self-analysis experience, as reported in his autobiography. Table 1 provides a schematic representation of the connections between Jung’s assertions concerning active imagination and Sandplay Therapy. For example, I highlighted that in both processes, it is the unconscious deciding what to depict (rather than the consciousness), while the consciousness lends its expressive means to the unconscious. As another important convergence, in both analytic processes, the resulting image is determined from both the consciousness and the unconscious and related to the person’s conscious situation.

In sum, Sandplay Therapy is an effective method to portray contents that are inexpressible through words and to favor individuation. Furthermore, apart from presenting many similitudes with active imagination, it can also contribute to the development of active imagination. Sandplay Therapy strengthens the imaginative capacity in front of the analyst, thereby preparing for the subsequent, independent, imaginative activity [8]. Moreover, I suggest that Sandplay Therapy might be used to practice active imagination in a “facilitated” and protected setting. The analysands are eased into letting the unconscious come up and emptying the mind from the Ego’s thought processes as the therapist provides them with evocative stimuli (i.e., the sandtray and the objects/symbols on the shelves) for their unconscious. Moreover, the patients objectify their unconscious by creating a three-dimensional image in the sandtray. Therefore, they can use this image as the starting point of active imagination. Usually, once the analysands have finished creating their image in the sandtray, they contemplate it. Hence, the therapist might prompt them to focus on the image, fertilizing it, and, once alive, to actively relate with the image by (inwardly) talking and interacting with the figures in the sandtray. Finally, they can use other means of objectification (e.g., a painting) to give form to what arose from the active imagination originated by the image in the sandtray and practice another active imagination experience, starting this time from the new objectified image. Thus, Sandplay Therapy might be used as a tool for helping to let the unconscious come up (while creating the image in the sandtray), but also, through the contemplation of the image created in the sandtray, as a means to foster the confrontation between the unconscious and the consciousness.

It is essential to highlight that the prompts that the analysts might offer to the analysands to practice active imagination from the image created in the sandtray must be provided within the Jungian/Kalff psychoanalytic approach, therefore avoiding the directiveness that might be used in sandtray therapy (e.g., gestalt techniques). Jung was generally non-directive but acted as a mentor in some cases, as he believed that his suggestions had an effect only if the patient was predisposed to them [7]. Along the same line, the analysts might prompt the patients to engage in active imagination using the image created in the sandtray but without giving specific suggestions or comments and without insisting that the patients try the experience if they do not want to.

**Figure 1:**
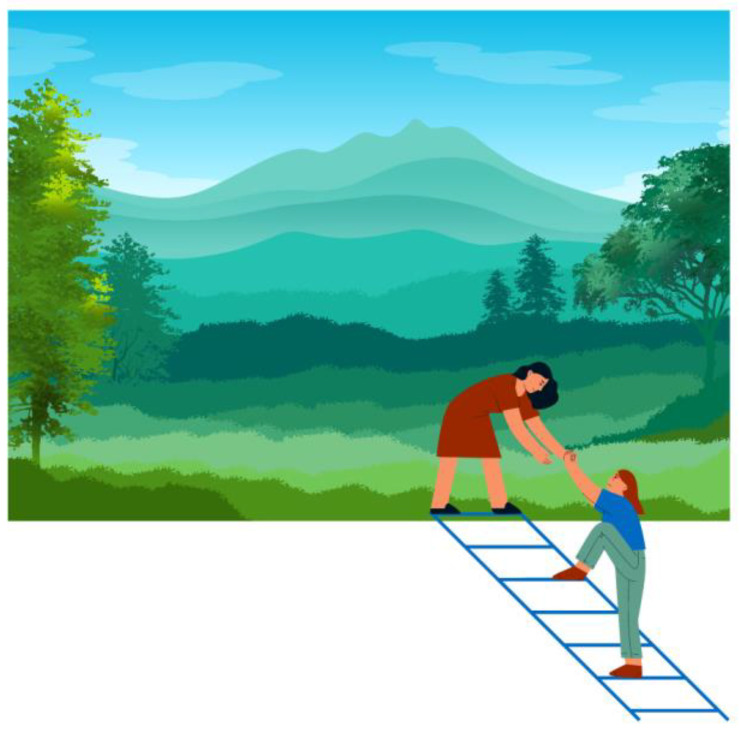
The dialogue between the unconscious and the consciousness. Note: Image created by the author with Canva.

In conclusion, Sandplay Therapy activates the dialogue between the unconscious and the consciousness in the analysand, as occurs during active imagination. Figure 1 depicts this contact, where the Ego (i.e., the person outside the landscape) is preparing to enter the scene through the help of the unconscious (illustrated by the person inside the landscape). Access into the landscape is allowed by a ladder, representing both the difficulties that the Ego may encounter in the early stages of its confrontation with the unconscious and the support coming from the unconscious itself that, in fact, extends its hand to help the Ego up. If consciousness and unconscious collaborate, the Ego can enter the scene and dialogue with the figures present in it; for example, the woman representing the unconscious could be the personification of the *Anima*. If we let our attention further fertilize the image (as suggested by Jung), we could also see much more. For example, we could see an analysand who is about to enter the image produced in the sandtray, supported by his/her unconscious or the analyst.

## Figures and Tables

**Table 1:** Connections between Jung’s assertions concerning Active Imagination (AI) and Sandplay Therapy (SPT).

Jung’s Writing on Active Imagination	(Kalff) Sandplay Therapy
“Often the hands know how to solve a riddle with which the intellect wrestled in vain” ([13], par. 180)	The “manifesto” of SPT as it is based on a significant involvement of the hands in the analytic process.
When there is a high blockage at the conscious level, only the hands are capable of fantasy [14]	In SPT, hands have a primary role—they shape the sand, choose the objects, and place them in the sandtray.
The hands draw or model images that are sometimes alien to the conscious mind [14]	The hands, in SPT, often give life to images beyond the consciousness’ control.
It is not the consciousness determining what to depict but rather the unconscious [6,13,16]	In SPT, the consciousness (through the hands, the sandtray, and the objects) loans its expressive tools to the unconscious content.
The product of AI is determined by both the consciousness and the unconscious [13]	The symbols used in SPT (i.e., miniatures, objects, and figures created in the sand) represent the third element originating from the confrontation between the consciousness and the unconscious.
The product of AI is related to the conscious situation of the person [17]	The symbols used by the person in SPT might have a relationship with the conscious situation of the person.
Patients try with all their energies to give shape to the unspeakable [20]	SPT allows for the representation of contents that are not verbally expressible.
The differentiation between the consciousness and the contents of the unconscious occurs through the isolation of the unconscious by its personification [21]	Through the three-dimensionality provided by miniatures, the manipulation of the sand, and the use of the tray’s blue space, the person is facilitated in personifying the unconscious content.
It is fundamental that the person lives the images instead of understanding and interpreting them [22]	Thanks to the observation/contemplation of the three-dimensional image in SPT, the person might activate the dialogue between the consciousness and the unconscious and live the image.
Archetypes must be objectified to avoid their over-flooding of the consciousness [3]	The sandtray—simultaneously a free and protected space [11] —limits the risk of inflation by the unconscious. The miniatures and the figures in the sand, objectifying an image arising from the unconscious, also serve this purpose.
It is important “to do” rather than just talk [20]	In SPT, the analysands “do”; they move with their whole body around the shelves and use their hands while placing, removing, or moving objects in the sandtray.
Creating a form allows the person to continuously study all its parts, favoring the development of the image’s potential to its maximum [20]	Thanks to the production of the image in the sandtray, the person is stimulated to observe the image from various angles, fueling the development of its individuation potential.
If the person meditates long enough upon a dream, carrying it inside him/herself and looking at it several times, something almost always emerges [20]	In SPT, after creating the image, the person observes it in a meditative/spiritual state. Furthermore, analysands carry the image within them and can continue to observe and contemplate it inwardly.
When the person concentrates on an internal image and does not interrupt its natural flow, the unconscious produces a series of images that make up a complete story [3]	SPT, through a series of images created in the sandtray across different sessions, narrates the psychic development that turns towards the individuation of the analysand.
Interpretations should be kept to a minimum, they should never go beyond the confines of the image produced by the analysand, and they should be interspersed with “maybes”, “ifs”, and “buts” [16]	The SPT analyst must interpret for him/herself the symbols emerging in the sandtray, generally without communicating them.
Jung, while waiting to build the altar in the village he built, found a red stone in the shape of a pyramid and understood that it must be his altar [21]	In SPT, the person can feel “chosen by the object” on the shelf rather than “choosing the object”.
Once Jung placed the red stone on the altar, he remembered the underground phallus of his dream as a child and felt relief [21]	In SPT, placing an object (or creating a shape) in the sandtray might activate unconscious elements and complexes and therefore a sense of relief.
It is essential to discover the hidden image behind an emotion [21]	SPT allows the person to observe (in an objectified form) the images emerging from the unconscious, with the consequent uncovering of the image hidden behind the emotion.

## Data Availability

Not applicable.

## References

[1] Jung C.G., Chodorow J. (1940). The psychological aspects of the Kore. Jung on Active Imagination.

[2] de Luca Comandini F., de Luca Comandini F., Mercurio R. (2002). Immaginazione attiva. Senso interno e valenze sociali dell’individualità psicologica [Active immagination. Inner meaning and social valences for the psychological individuation]. L’immaginazione Attiva [Active Imagination].

[3] Jung C.G., Chodorow J. (1935). The Tavistock lectures. Jung on Active Imagination.

[4] Jung C.G., Chodorow J. (1947). Three letters to Mr O. Jung on Active Imagination.

[5] Loscalzo Y. (2024). Active imagination: A thorough presentation of a method applicable across psychodynamic approaches. Cult. Psychol..

[6] Jung C.G., Chodorow J. (1933/1950). A study in the process of individuation. Jung on Active Imagination.

[7] Chodorow J., Chodorow J. (1997). Introduction. Jung on Active Imagination.

[8] Carducci P., de Luca Comandini F., Mercurio R. (2002). Immaginazione attiva e Sandplay Therapy [Active imagination and Sandplay Therapy]. L’immaginazione Attiva [Active Imagination].

[9] Garufi B., de Luca Comandini F., Mercurio R. (2002). La poesia [The poem]. L’immaginazione Attiva [Active Imagination].

[10] Lowenfeld M. (1979). The World Technique.

[11] Kalff D.M. (1996). Il Gioco Della Sabbia e la Sua Azione Terapeutica Sulla Psiche. A Cura e Con Introduzione di Marco Garzonio [Sand Play and Its Therapeutic Action on the Psyche. Edited and with introduction by Marco Garzonio.

[12] Kalff M. (2018). Ascoltando il Corpo. Nuove Vie Per il Gioco Della Sabia [Listening to the Body. New Ways for Sandplay].

[13] Jung C.G., Chodorow J. (1916/1958). The transcendent function. Jung on Active Imagination.

[14] Jung C.G., Chodorow J. (1929). Commentary on The Secret of the Golden Flower. Jung on Active Imagination.

[15] Marinucci S., Marinucci S., D’Andreamatteo D. (2016). La Sandplay Therapy di Dora Kalff [The Sandplay Therapy of Dora Kalff]. Giocare Con la Sabbia. Versatilità e Prospettive di un Metodo di Cura [To Play with the Sand. Versalities and Perspectives of a Treatement Method].

[16] Jung C.G., Chodorow J. (1947). On the nature of the psyche. Jung on Active Imagination.

[17] Jung C.G., Chodorow J. (1955). Mysterium Coniunctionis. Jung on Active Imagination.

[18] Montecchi F. (2016). I Disturbi Alimentari Nell’infanzia e Nell’adolescenza. Comprendere, Valutare e Curare. Nuova Edizione [Eating Disorders in Childhood and Adolescence. To Understand, Evaluate, and Treat. New Edition].

[19] D’Andreamatteo D., Marinucci S., D’Andreamatteo D. (2016). Aspetti tecnici della Sandplay Therapy [Technical aspects of Sandplay Therapy]. Giocare Con la Sabbia. Versatilità e Prospettive di un Metodo di Cura [To Play with the Sand. Versalities and Perspectives of a Treatement Method].

[20] Jung C.G., Chodorow J. (1931). The aims of psychotherapy. Jung on Active Imagination.

[21] Jung C.G., Jaffé A. (1961). A confronto con l’inconscio [Confrontation with the unconscious]. Ricordi, Sogni, Riflessioni [Memories, Dreams, Reflection].

[22] Jung C.G., Chodorow J. (1928). The technique of differentiation between the ego and the figures of the unconscious. Jung on Active Imagination.

[23] Kalff M. (2007). Twenty-one points to be considered in the interpretation of a sandplay. J. Sandplay Ther..

[24] Adorisio A., de Luca Comandini F., Mercurio R. (2002). La danza e il movimento [The dance and the movement]. L’immaginazione Attiva [Active Imagination].

[25] Garzonio M., Kalff D.M. (2022). Introduzione all’edizione italiana. Dora Kalff: La polvere, il vasaio, la nuova creatura e lo spirito riparatore [Introduction to the Italian edition. Dora Kalff: The dust, the potter, the new creature, and the repairing spirit]. Il Gioco Della Sabbia e la sua Azione Terapeutica Sulla Psiche. A Cura e con Introduzione di Marco Garzonio [Sand Play and Its Therapeutic Action on the Psyche. Edited and with Introduction by Marco Garzonio].

